# Flashing Lights, Dark Shadows, and Future Prospects of the Current European Legislation for a Better Traceability and Animal Health Requirements for Movements of Small Animal Germinal Products

**DOI:** 10.3389/fvets.2022.852894

**Published:** 2022-05-09

**Authors:** Michela Pugliese, Salvatore Monti, Vito Biondi, Gabriele Marino, Annamaria Passantino

**Affiliations:** Department of Veterinary Sciences, University of Messina, Messina, Italy

**Keywords:** semen, oocytes, embryos, dog, cat, European legislation

## Abstract

Recently, there has been an increasing movement of germinal products of dogs (*Canis lupus familiaris*) and cats (*Felis silvestris catus*) between the Member States. Therefore, Europe laid down and harmonized rules on the marking of straws and other packages containing germinal products [Commission Delegated Regulation (EU) 2020/686]. Given that germinal products' movement may increase the risk of infectious disease spread, requirements regarding animal health have been revised focusing on control of rabies and echinococcosis, although there are new emerging diseases that may require, even locally, specific requirements. For this reason, veterinarians, operators, and official veterinarians are involved in different phases of the process. Because non-veterinary operators can operate in all phases, they should have a limited role in collecting germinal products, especially for feline species. Veterinarians, instead, should have a main role in the health evaluation of donors, in collecting germ cells with medical techniques and in depositing sperm and embryos with endoscopic or surgical methods. The official veterinarians are the main ones responsible for the application of the rules. This paper aims to provide an overview of the European legislative framework regarding the newly delegated regulation on germinal products in small animals (dogs and cats), highlighting some of the benefits and critical aspects regarding its functioning.

## Introduction

Germinal products (semen, oocytes, and embryos) collected or produced from animals for reproduction have always been marked and distributed worldwide. Given that these products are widely used in the animal population, including pet animals, and are also moved within the European Union (EU), they—if not handled properly or not categorized with the correct health status—may represent an important risk for the spread of animal diseases, causing poor animal welfare and substantial economic losses (1). To prevent the aforesaid risk, EU has recently adopted with Commission Delegated Regulation (EU) 2020/686 (hereinafter EU Reg.) (2) specific procedures on the traceability of germinal products of kept terrestrial animals, including dogs (*Canis lupus familiaris*) and cats (*Felis silvestris catus*). Additionally, the European legislator has been established rules on the marketing of straws and other packages containing such canine and feline germinal products. Aims of the present regulation have been preventing and managing risks from infectious diseases, guarantying a high level of protection of animal health and consequently promoting animal welfare. Indeed, preventive pathway management, based on health standards and biosecurity strategies, is the basis of that.

Given these considerations, the Authors carry out preliminarily a succinct narrative review of the contents of the current European legislation on pets' germinal products, then express their opinion emphasizing the good and bad points—lights and shadows—that characterize its application.

## The Legislative Framework in the European Community (Hard Law)

Over the past 5 years, the EU has developed policies regarding germinal products.

The first rules were originally laid down in Directives 88/407/EEC (3), 89/556/EEC (4), 90/429/EEC (5), and 92/65/EEC (6) and they concerned bovine, ovine, caprine, porcine, and equine species. The previous framework was a collection of individual pieces of legislation and concerned only a few species. To consolidate and modernize the existing law, all these directives were replaced by Regulation (EU) 2016/429 (7), so-called “Animal Health Law, AHL”, that lays down rules on transmissible animal diseases, and for the registration and approval of germinal products establishments and, traceability of consignments and the animal health requirements for germinal products moved within the Union. Successively, this regulation was supplemented by the EU Reg. to recognize germinal product establishments and the traceability and animal health requirements for movements within the EU of germinal products also of other animals, such as that of dogs and cats. It has also been adapted to enable rapid responses to outbreaks of emerging diseases.

All these rules are strongly linked, and many are applied in tandem.

### Commission Delegated Regulation (EU) 2020/686

The EU Reg. requires that all germinal products (semen, oocytes, and embryos) of dogs and cats, before each transfer between Member States (MSs), must be identified. For more details in Box 1 are reported articles that provide rules and requirements relating to dogs and cats.

Box 1 **Article relating to rules and requirements for movements within the Union of canine and feline germinal products.**

In Article 11: Traceability requirements

In Article 36: Animal health requirements

In Articles 39(1) and 40: Rules concerning animal health certification

In Articles 41, 42 and 43: Rules on notification.

Operators^1^, as stated in Article 11, shall mark each straw or other package in which germinal products, whether separated into individual doses, are placed, stored, and transported. On each straw and/or package following information must be reported: (i) the date of the collection or production of germinal products; (ii) the species and identification of the donor animal or all animal donors (in the case of a single straw or another package contains semen collected from multiple animal donors); (iii) the address of the establishment^2^ of collection or production, processing and storage of germinal products, or the registration or approval number if previously assigned, including the ISO 3166-1 alpha-2 code of the country. If it is exported sex-sorting of semen at an establishment other than the establishment of its collection or production, shall be reported any info to identify the establishment where that semen was sex-sorted. If the semen is frozen in pellets, the operator may mark the goblet containing semen pellets of a single donor instead of marking each pellet in that goblet.

Information on the traceability requirements for germinal products of dogs and cats are contained in Chapter 3 of Part II of the EU Reg. Germinal products collected from pets, as referred to in Article 36, can be only moved to other MSs if these animals have been born and remained since birth in the UE or have entered the Union following the requirements for entry into the UE. These animals, marked by the implantation of a transponder or by a readable tattoo enshrined in Article 17 of Regulation (EU) 576/2013 (8) of the European Parliament and the Council or identified following Article 70 of the Regulation (EU) 2019/2035 (9), not must show clinical signs of disease on the day of collection and must come from an establishment where the infection with rabies virus has not been confirmed for at least 30 days before the date of collection of germinal products. All animals must have received an anti-rabies vaccination and comply with any preventive health measure for diseases or infections other than rabies set out in Part 2 of Annex VII to the Delegated Regulation (EU) 2020/688 (10). A further requirement which shall be fulfilled regards the not use for natural breeding during at least 30 days before the date of collection. On the other hand, as regards the movement of germinal material between confined establishments, in addition to what has already been said, these will only be possible if the animals come from an establishment in which no category D disease has been reported, such as rabies and echinococcosis, for which it is necessary to implement prevention plans (Art.1, Regulation EU 2018/1882) (11), for at least 30 days before the date of collection and are identified and registered following the rules of the confined establishment. Furthermore, these animals must be subjected to clinical examination by the establishment veterinarian liable for the activities carried out at the confined establishment, without showing any signs of the disease on the day of collection of the sperm, oocytes, or embryos. As stated in Article 39, before signing an animal health certificate for movements between MSs of consignments of germinal products of dogs or cats, the official veterinarian shall carry out (i) a visual examination of the transport container to check the seal and number applied by the operator on the transport container or if necessary, the germinal products placed in the transport container and, to seal and number the transport container after that check; (ii) a documentary check of the data submitted by the operator to ensure that the information to be certified is supported by the records kept at the establishment; (iii) the mark on the straws or other packages corresponds with the number provided in the animal health certificate and on the container in which they are transported; (iv) the requirements referred in Article 36 have been fulfilled. The official veterinarian shall carry out the checks and examinations and, issue the animal health certificate within 72 h preceding the time of dispatch of the consignment of germinal products. The animal health certificate shall be valid for 10 days from the date of issuing. The minimum information contained in the health certificate is given by annex IV of the regulation and presented in Box 2. Where consignments of germinal products of dogs or cats are moved to another MSs, the operator shall notify the competent authority of the MS of origin of the consignments in advance of the intended movement of consignments of germinal products, prescribed by Article 41. Operators required to notify the competent authority of the MS of origin of the consignments shall provide that competent authority with the information concerning each consignment of germinal products to be moved to another MS provided for in point 2 (a)–(f) of Annex IV (Article 42). In the event of power cuts and other disturbances of Information Management System for Official Controls (IMSOC), the competent authority of the place of origin of the consignment of germinal products of dogs or cats, to be moved to another Member State, shall notify the Commission and the competent authority of the place of destination of the movement of that consignment by fax or email (see article 43). The notification referred to in paragraph 1, shall be carried out by the competent authority of the place of origin of the consignment of the germinal products following the contingency arrangements to be applied in the event of unavailability of any of the functionalities of IMSOC.

Box 2 **Information to be contained in the animal health certificate.**

Name and address of the consignor and the consignee

Name and address of the establishment of dispatch, and the unique registration number, where the establishment of dispatch was assigned with such registration number; or the unique approval number of that confined establishment, where the establishment of dispatch is a confined establishment;

Name and address of the establishment of destination and, where the establishment of destination is a confined establishment, the unique approval number of that confined establishment;

Type of germinal products and the species of donor animals;

Number of straws or other packages to be dispatched;

Information allowing identification of germinal products: the species, where necessary the subspecies, and identification of the donor animals from which germinal products were collected; the marking applied to the straws or other packages in accordance with Article 11; the place and date of their collection or production; the number on the seal applied to the transport container; the information on the animal health situation, additional guarantees and, where necessary, test results in relation to the Member State or zone thereof; (the establishment of origin of the donor animals; the donor animals from which germinal products were collected; the germinal products to be dispatched);

- Date and place of issue of the animal health certificate, the name, capacity and signature of the official veterinarian, and the stamp of the competent authority of the place of origin of the consignment.

For a better overview, are reported in Box 2 the articles that provide rules and requirements relating to dogs and cats.

### Commission Implementing Regulation (EU) 2021/403

This regulation concerns animal health/official certificates (hereinafter referred to as “the certificates”) that should accompany consignments of germinal products of certain categories of terrestrial animals, including dogs and cats, moved to other MSs or for the entry into the Union (12). The certificates should be issued following model GP-CANIS-FELIS-INTRA (Art. 13, point e) set out in Annex I. The use of this model certificate is started on October 18, 2021. The certificates contain details of the consignment and, specific animal and public health information, as well as animal welfare information, where necessary, certified by the official veterinarian. In the case of movements between MSs, certificates contain a part designated to draw up a record of official controls performed during such movements and at the place of destination, as well as the results of those official controls.

## Soft Law

### OIE (Office International des Epizooties)

Animal health measures have increased the importance to facilitate safe international trade of animals and animal products, including germinal products. Based on that, the Agreement on the Application of Sanitary and Phytosanitary Measures (SPS-Agreement) has encouraged the members of the World Trade Organization (WTO) to base their sanitary measures on international standards, guidelines, and recommendations, where they exist. OIE, which is the WTO reference organization for standards relating to animal health and zoonosis, has published two codes (Terrestrial and Aquatic) and manuals as the principal reference for WTO members. Member countries of OIE can utilize the standards to protect themselves from infectious diseases, providing this does not involve sanitary barriers that cannot be justified. To facilitate the global distribution of semen free from specific pathogenic organisms, the Terrestrial Animal Health Code (chapter 4.6–4.12) reports specific standards but concerning only zootechnical animals (13).

### International Breeding Regulations of the Federation Cynologique Internationale

Federation Cynologique Internationale (FCI) breeding regulations are directly applied to all FCI member countries (no. 42 full European members) and contract partners (no. 2 European partners) (http://www.fci.be/en/members/members.aspx?section=3). In these member countries, the breeding should be carried out with pedigree dogs, a sound temperament, healthy in functional and hereditary terms and registered with a studbook or register recognized by the Federation.

Section no. 13 regards artificial insemination. It is reported that in the case of the female dog is to be artificially inseminated, the veterinarian is the only authorized person to collect semen in the dog and to provide a certification in which the identification of the stud dog is guaranteed. The veterinarian that takes the semen and performs the artificial insemination, emits a second certification, including the place and date of the insemination, the name and studbook registration number of the female dog and, the name and address of the owner of the female dog. The owner of the stud dog from which the semen was collected must provide a signed stud service certification to the owner of the female dog in addition to the veterinary certification.

The collected semen is legally considered property. When the semen is collected for processing the ownership of the semen needs to be specified by a written document that should also indicate the time of collection, the amounts of sperm, the identification of amounts, the place of storage and the identification of the stud dog. It is strongly recommended to perform a DNA profile from every dog for which the semen is deposited. When the stud dog is sold, the owner must provide information about the already collected frozen semen to the other party.

The semen can only be used in national policies for mating are satisfied, particularly guaranteeing that the semen may only be used for female dogs recorded in the FCI-recognized studbooks.

The more restrictive soft law comes from the French Kennel Club, which registers litters if the insemination is performed by a veterinarian who has followed a special course at one of the veterinary schools in Lyon, Maisons-Alfort or Nantes. Imported semen must be kept in an official dog semen bank before being sent to the inseminating veterinarian and banks are in the three veterinary schools mentioned above (14).

### International Feline Federation Breeding Rules

The Federation Internationale Feline (FIFe) includes 42 members of 40 countries (http://www1.fifeweb.org/wp/org/org_mem.php). In these member countries, the breeding should be carried out with pedigree cats. A male cat shall have a veterinary certificate confirming that both testicles are normally descended into the scrotal sac. No rules have been established for sperm collection, shipment and use for artificial insemination.

### Responsible Dog/Cat Breeding Guidelines

The guidelines of responsible dog and cat breeding are available in the EU platform of animal welfare (https://ec.europa.eu/food/animals/animal-welfare/eu-platform-animal-welfare_en) and maybe listed among the soft law of the topic. They establish that only manual collection of sperm is accepted, among methods, excluding categorical electroejaculation. Semen collection (and artificial insemination) must only be performed by a suitably qualified veterinarian, competent and authorized in the practice of the method.

## Lights and Shadows of the EU Regulation

The application of EU Reg. shows some flashing lights and a few dark shadows. Generally, hard law has provided various relevant advantages. The main flashing light is related to movements' notification of germinal products between MSs. In notifying this, it is given the possibility of ensuring their traceability in situations where these movements may be linked to a risk of spreading transmissible animal diseases. For this motivation, the European legislator has laid down rules on the registration of establishments keeping animals or handling germinal products or transporting them. In doing so, the competent authority will be able to perform adequate surveillance and to prevent, control and eradicate transmissible animal diseases.

Another element taken into consideration to ensure that the animal health conditions for the transport of germinal products are not compromised is the sealing of containers in which germinal products are transported. An official veterinarian should certify the consignment of germinal products and verify the content of the transport container. In the case that the official veterinarian breaks the seal, he/she should later re-seals the transport container.

These specific surveillance requirements and targeted movement of consignments of germinal products should provide for a sufficient guarantee to prevent the spread of animal diseases.

Article 11 of the EU Reg. establishes that operators shall “mark the straw”. There is a custom to dispatch the semen in containers that are not marked or simply written with a (more or less) indelible pen. Although the information can be detected from the certificates, the law requires that even in the absence of certificates, the minimum information of the donor animal and the establishment must be traceable and legible on the straw. Many establishments do not receive germinal products in pen-written straws. It is recommended that the information should therefore be marked with a special indelible ink printer. There are several models on the market and generally, companies can supply pre-marked straws on request.

To the risk of infectious diseases spread, one of the main news of the EU Reg. is that the pet is vaccinated against the rabies virus (art. 36). This can be considered another advantage. The reason for that, in the opinion of the Authors, is related to the possibility of venereal rabies transmission (15). WHO and its partners aimed to eradicate rabies within 2030 (16). Human Lyssavirus infections are reported in EU countries, in fact for 2019, four human cases of travel-related rabies were reported in Italy, Latvia, Spain, and Norway following an exposure in Tanzania, India, Morocco, and the Philippines, respectively (17). One locally acquired fatal case of European bat lyssavirus (EBLV-1) infection was reported in France. EU includes countries classified such as no risk countries, low-risk countries (Bulgaria, Croatia, the Czech Republic within 50 km border Poland/Slovakia, Hungary, Latvia, Slovakia, Slovenia) and high-risk countries (Albania, Armenia, Azerbaijan, Bosnia and Herzegovina, Congo, Kosovo, Lithuania, Montenegro, Poland, Romania, Russian Federation, Serbia, Turkey, Ukraine). This is the basis that let the EU adopt strict measures and promote the use of vaccines. The period of validity of the vaccination starts from the establishment of the protective immunity, which shall not be <21 days from the completion of the vaccination protocol required by the manufacturer for the primary vaccination and continues until the end of the period of protective immunity—as prescribed by current legislation—although some countries as Denmark require at least 30 days (14). The period of validity of the vaccination is indicated by an authorized veterinarian or an official veterinarian in the appropriate part of the identification document. As result, the licensed validity ranges from 1 to 3 years. Although in some countries, 3-years lasting vaccines are available and included in local rules, other countries, as Denmark, requires a vaccination not more than 12 months before the collection of semen (14). Considering that the evidence-based duration of the new generation of rabies vaccines is at least 3 years, vaccination schedules and countries' requirements need to agree toward a 3-years indication.

Another positive aspect of the EU Reg. was to indicate some soft indications for echinococcosis that represent a substantial disease burden. The larval stage of *Echinococcus multilocularis* is the etiological agent of alveolar echinococcosis, a parasitic zoonotic disease with an estimated 17.400 new infections/year (18). The life cycle of *E. multilocularis* involves small rodent intermediate hosts, such as arvicolids and, depending on the epidemiological settings, wild or domestic canid definitive hosts. Parasite eggs are transmitted to intermediate hosts *via* carnivore feces, whose distribution in the environment is driven by the defecating behavior of the final hosts (19). The transmission of eggs with semen, oocytes or embryos has never been demonstrated. However, in the opinion of the Authors, eggs may be present in preputial skin and saliva and dogs commonly lick genitalia before or after mating. In this EU Reg. the preventive measures are provided in Annex VII. It establishes that the risk-mitigating measures for infestation with *Echinococcus multilocularis* are those laid down in Commission Delegated Regulation (EU) 2018/772 (20) in association with Commission Implementing Regulation (EU) 2018/878 (21). The first one indicates that the treatment against *E. multilocularis* shall be carried out within not more than 120 h and not <24 h before the time of the germinal product scheduled entry into the territory or parts of the territory of such MS. The treatment shall be administered by a veterinarian and shall consist of a medicinal product that contains the appropriate dose of praziquantel, or other pharmacologically active substances, which alone or in combination, have been proven to reduce the burden of mature and immature intestinal forms of the *E. multilocularis* parasite in dogs at least as effectively as praziquantel. The Regulation 2018/878 (21) gives a list of the countries that require preventive treatment, at the date: Ireland, Finland, and Malta.

In the case of dogs affected by other diseases than rabies and echinococcosis, preventive health measures are applicable and adopted following Article 19 (1) of Regulation (EU) 576/2013 (8). It establishes that where preventive health measures are necessary for the protection of public health or the health of pet animals to control diseases or infections that are likely to be spread due to the movement of those pet animals, the Commission shall be empowered to adopt delegated acts concerning species-specific preventive health measures for such diseases or infections. In this way, some MSs laws and soft laws give requirements for brucellosis and leptospirosis. A blood test for *B. canis, Leptospira canicola*, and *L. ichterohaemorrhagica* is required for the semen importation in Austria (A), Czech Republic (CR), and Sweden (S). The test should be performed not earlier than 20 days the collection of semen (S and CR only for frozen semen), or 15 days (S), at the time of the semen collection (A), after 14 days (A), not later than 30 days after the collection of semen (S and CR only for frozen semen). In Norway, the dog must be vaccinated against leptospirosis within 365 days before the semen collection (14).

On the other hand, a few shadows dark the horizon of the EU Reg. and they concern the different professional figures (veterinarians, operators, and official veterinarians) that involve in phases of the process of semen collection, handling and transferring. In the Authors' opinion, non-veterinarian operators have a limited role in collecting, evaluating, and packaging the germinal plasma, especially for feline species. There is a tendency in different countries to refer to operators different from veterinarians. This is partially acceptable for farm animals. First, it should be ensured that any member of staff handling dogs and cats during all steps of the artificial insemination has completed a training course recognized by the competent authorities. Therefore, training should be a prerequisite for any person handling animals, and training should be provided only by organizations approved by the competent authorities. In turn, the competent authority should ensure that the staff is duly trained by issuing a certificate of competence. Diagnostic tests, treatments or vaccinations for rabies, echinococcosis, brucellosis, and leptospirosis are performed under veterinarian supervision. The veterinarians must also certify that the donor did not show disease symptoms on the day of collection. Many diseases may affect the quality of the germ cell collection and spread eventual germs. A non-exhaustive list includes the fever, infection at genital or urinary levels, diarrhea. A general recommendation is the certification of normal testicles in the male donor, to avoid the spread of genetic defects, as cryptorchidism (14). In this view, wider genetic screening may be required by breed association or by the market (1). The semen collection in the dog is performed with manual stimulation with or without an artificial vagina. As in other species, it is not considered a medical act and it may be performed by trained operators, although FCI recommends that veterinarians are firstly involved in this technique. Furthermore, in some EU countries, like Sweden and Denmark, only veterinarians may collect semen (14). Otherwise, the feline semen is collected under deep sedation or with electroejaculation (22), these techniques are not included in the responsible cat breeding guidelines. Manual collection of sperm in cats is ineffective. In the Authors' opinion, to guarantee animal welfare and for the use of anesthetics, it is undoubted that these techniques are performed only by a veterinarian. The same consideration for *in vivo* oocyte and embryo recovery, that in these species are performed surgically.

Instead, germ cell manipulation, evaluation and certification may be performed by many trained operators, including veterinarians, biotechnologists, biologists, technicians.

The French Kennel Club states that imported semen must be kept in an official dog semen bank in one of the veterinary schools of Lyon, Maisons-Alfort or Nantes. In other countries, germ banks may be managed by formed operators and even insemination does not require specific authorization. Artificial insemination in dogs is performed with a vaginal or intrauterine deposit of the semen (23). Poorly motile chilled semen and frozen semen should be deposited in the uterus to improve the pregnancy rate. Feline insemination is always performed with an intrauterine technique for the bad characteristics of the material in terms of the number of sperm, volume, motility, and abnormalities (24). The surgical intrauterine deposit is not ethically accepted (responsible cat/dog breeding guidelines) considering that the endoscopic route is an alternative and efficient technique. The endoscopic or even surgical deposition of semen, ova and embryo are all veterinarians' skills.

## Conclusion

EU Reg. is the first European law that harmonizes the rules on canine and feline germ cell movements in MSs. It gives important technical indications and provides the minimal contents of the certificate. It distinguishes the role of official veterinarians from operators that collect and handle the semen. Semen collection and artificial insemination in dogs and cats is not always a simple zootechnical technique. In the Authors' opinion it requires trained operators and in most cases veterinarian operators, in agreement to FCI and other soft laws.

## Author Contributions

AP: conceptualization and supervision. GM: methodology. MP: validation. SM: investigation. VB: data curation. GM and SM: writing—original draft preparation. MP and AP: writing—review and editing. All authors contributed to the article and approved the submitted version.

## Funding

This research has been supported by a grant from a FEASR-PSR Sicily 2014–2020, Misura 10, Sottomisura 10.2 b, CUP G49J21003940009.

## Conflict of Interest

The authors declare that the research was conducted in the absence of any commercial or financial relationships that could be construed as a potential conflict of interest.

## Publisher's Note

All claims expressed in this article are solely those of the authors and do not necessarily represent those of their affiliated organizations, or those of the publisher, the editors and the reviewers. Any product that may be evaluated in this article, or claim that may be made by its manufacturer, is not guaranteed or endorsed by the publisher.

## Footnotes

^1^The operator is defined by the Regulation (EU) 2016/429 (7), point 24 of article 4, as “any natural or legal person having animals or products under his responsibility, including for a limited duration of time, but excluding pet keepers and veterinarians”.

^2^Establishment means any premises, structure, or in the case of open-air farming, any environment or place, where animals or germinal products are kept, on a temporary or permanent basis, except for: (a) households where pet animals are kept; (b) veterinary practices or clinics.

## References

[1] Quartuccio M Biondi V Liotta L Passantino A. Legislative and ethical aspects on use of canine artificial insemination in the 21st century. Ital J Anim Sci. (2020) 19:630–43. 10.1080/1828051X.2020.1775503

[2] Commission Delegated Regulation (EU) 2020/686 of 17 December 2019 supplementing Regulation (EU) 2016/429 of the European Parliament and of the Council as regards the approval of germinal product establishments and the traceability and animal health requirements for movements within the Union of germinal products of certain kept terrestrial animals (Text with EEA relevance). Text with EEA relevance. OJ. (2020) 174:1–63.

[3] Council Directive 88/407/EEC of 14 June 1988 laying down the animal health requirements applicable to intra- Community trade in and imports of deep-frozen semen of domestic animals of the bovine species. OJ. (1988) 194:10–23.

[4] Council Directive 89/556/EEC of 25 September 1989 on animal health conditions governing intra-Community trade in and importation from third countries of embryos of domestic animals of the bovine species. OJ. (1989) 302:1–11.

[5] Council Directive 90/429/EEC of 26 June 1990 laying down the animal health requirements applicable to intra- Community trade in and imports of semen of domestic animals of the porcine species. OJ. (1990) 224:62–72.

[6] Council Council Directive 92/65/EEC of 13 July 1992 laying down animal health requirements governing trade in and imports into the Community of animals semen ova and embryos not subject to animal health requirements laid down in specific Community rules referred to in Annex A (I) to Directive 90/425/EEC. OJ. (1992) 268:54–72.

[7] Regulation (EU) 2016/429 of the European Parliament and of the Council of 9 March 2016 on transmissible animal diseases and amending and repealing certain acts in the area of animal health (‘Animal Health Law'). OJ. (2016) 84:1–208.

[8] Regulation (EU) No 576/2013 of the European Parliament and of the Council of 12 June 2013 on the non-commercial movement of pet animals and repealing Regulation (EC) No 998/2003 Text with EEA relevance. OJ. (2013) 178:1–26.

[9] Commission Commission delegated regulation (EU) 2019/2035 of 28 June 2019 supplementing supplementing Regulation (EU) 2016/429 of the European Parliament and of the Council as regards rules for establishments keeping terrestrial animals and hatcheries and and the traceability of certain kept terrestrial animals and hatching eggs. Text with EEA relevance. OJ. (2019) 314:115.

[10] Commission Commission Delegated Regulation (EU) 2020/688 of 17 December 2019 supplementing Regulation (EU) 2016/429 of the European Parliament and of the Council as as regards animal health requirements for movements within the Union of terrestrial animals and hatching eggs. OJ. (2020) 174:140–210.

[11] Commission Commission Implementing Regulation (EU) 2018/1882 of 3 December 2018 on the application of certain disease prevention and control rules to categories of listed diseases and establishing a list of species and groups of species posing a considerable risk for the spread of those listed diseases C/2018/7920. OJ. (2018) 308:21–9.

[12] Commission Commission Implementing Regulation (EU) 2021/403 of 24 March 2021 laying down rules for the application of Regulations (EU) 2016/429 and (EU) 2017/625 of the European Parliament and of the Council as regards model animal health certificates and model animal health/official certificates for for the entry into the Union and movements between Member States of consignments of certain categories of terrestrial animals and germinal products thereof official certification regarding such certificates and repealing Decision 2010/470/EU. OJ. (2021) 113:1–935.

[13] OIE. World Organization For Animal Health, Terrestrial Animal Health Code. (2021). Paris: OIE.

[14] Linde-Forsberg C. Regulations and recommendations for international shipment of chilled and frozen canine semen. In: Concannon PW England G Verstegen J Linde-Forsberg C, editors. Recent Advances in Small Animal Reproduction. Ithaca, NY: International Veterinary Information Service (2001).

[15] Offiong EEA Essien CA Otoh AJ Eyo GD Habib M Obioku OE. Transmission of rabies to a dog through the semen. Int J Dev Sustain. (2014) 3:956–8.

[16] Food Food Agriculture Organization of the United Nations World Organization for Animal Health World Health Organization Global Alliance for Rabies Control. Zero by 30: The Global Strategic Plan to End Human Deaths from Dog-Mediated Rabies by 2030. Geneva: WHO (2018). Available online at: https://apps.who.int/iris/bitstream/handle/10665/272756/9789241513838-eng.pdf?ua=1 (accessed November 3, 2021).

[17] Gossner CM Mailles A Aznar I Dimina E Echevarría JE Feruglio SL. Prevention of human rabies: a challenge for the European Union and the European Economic. Area Euro Surveill. (2020) 25:2000158. 10.2807/1560-7917.ES.2020.25.38.2000158 32975184 PMC7533618

[18] EFSA AHAW Panel (EFSA Panel on Animal Health and Welfare). Scientific opinion on Echinococcus multilocularis infection in animals. EFSA J. (2015) 13:4373. 10.2903/j.efsa.2015.4373

[19] Casulli A Thomas F Barth E Tamarozzi F. Echinococcus multilocularis. Trends Parasitol. (2019) 35:738–9. 10.1016/j.pt.2019.05.005 31182385

[20] Commission Delegated Regulation (EU) 2018/772 of 21 November 2017 supplementing Regulation (EU) No 576/2013 of the European Parliament and of the Council with regard to preventive health measures for the control of Echinococcus multilocularis infection in dogs and repealing Delegated Regulation (EU) No 1152/2011. OJ. (2018) 130:1.

[21] Commission Commission Implementing Regulation (EU) 2018/878 of 18 June 2018 adopting the list of Member States or or parts of the territory of Member States that that comply with the rules for categorization laid down in Article 2(2) and (3) of Delegated Regulation (EU) 2018/772 concerning the application of preventive health measures for the control of Echinococcus multilocularis infection in dogs. OJ. (2018) 155:1.

[22] Zambelli D Cunto M. Semen collection in cats: techniques and analysis. Theriogenology. (2006) 66:159–65. 10.1016/j.theriogenology.2006.01.054 16527340

[23] Mason SJ. Current review of artificial insemination in dogs. Vet Clin North Am Small Anim Pract. (2018) 48:567–80. 10.1016/j.cvsm.2018.02.005 29680455

[24] Zambelli D Cunto M. Transcervical artificial insemination in the cat. Theriogenology. (2005) 64:698–705. 10.1016/j.theriogenology.2005.05.020 15963560

